# Restoration of the patient-specific anatomy of the distal fibula based on a novel three-dimensional contralateral registration method

**DOI:** 10.1186/s40634-022-00487-7

**Published:** 2022-05-20

**Authors:** Anna-Katharina Calek, Sandro Hodel, Bettina Hochreiter, Arnd Viehöfer, Sandro Fucentese, Stephan Wirth, Lazaros Vlachopoulos

**Affiliations:** Department of Orthopedics, Balgrist University Hospital, University of Zurich, Forchstrasse 340, CH-8008 Zurich, Switzerland

**Keywords:** Distal fibula, Fibular malunion, 3D registration

## Abstract

**Purpose:**

Posttraumatic fibular malunion alters ankle joint biomechanics and may lead to pain, stiffness, and premature osteoarthritis. The accurate restoration is key for success of reconstructive surgeries. The aim of this study was to analyze the accuracy of a novel three-dimensional (3D) registration algorithm using different segments of the contralateral anatomy to restore the distal fibula.

**Methods:**

Triangular 3D surface models were reconstructed from computed tomographic data of 96 paired lower legs. Four segments were defined: 25% tibia, 50% tibia, 75% fibula, and 75% fibula and tibia. A surface registration algorithm was used to superimpose the mirrored contralateral model on the original model. The accuracy of distal fibula restoration was measured.

**Results:**

The median rotation error, 3D distance (Euclidean distance), and 3D angle (Euler’s angle) using the distal 25% tibia segment for the registration were 0.8° (− 1.7–4.8), 2.1 mm (1.4–2.9), and 2.9° (1.9–5.4), respectively. The restoration showed the highest errors using the 75% fibula segment (rotation error 3.2° (0.1–8.3); Euclidean distance 4.2 mm (3.1–5.8); Euler’s angle 5.8° (3.4–9.2)). The translation error did not differ significantly between segments.

**Conclusion:**

3D registration of the contralateral tibia and fibula reliably approximated the premorbid anatomy of the distal fibula. Registration of the 25% distal tibia, including distinct anatomical landmarks of the fibular notch and malleolar colliculi, restored the anatomy with increasing accuracy, minimizing both rotational and translational errors. This new method of evaluating malreductions could reduce morbidity in patients with ankle fractures.

**Level of evidence:**

IV

## Introduction

Ankle fractures are common injuries in adults and account for 7–10% of all fractures [1, 2]. The distal fibula fracture is the element most commonly involved in all patterns of ankle fractures [3]. If anatomic reduction cannot be achieved, distal fibular malunion, shortening and malrotation of the fibula, occurs. Since the fibula is the lateral buttress of the talus, malunion can lead to widening of the ankle mortise and talar instability [4]. Widening with a translation of the talus of 1 mm already reduces the contact area to 60% of the joint [5]. Thus, the biomechanics of the ankle joint are altered, which may result in pain, stiffness, and premature osteoarthritis [6].

Various osteotomies, which are usually performed freehand [7, 8], allow the correction of the malunited fibular fracture [9]. In more than 75% of patients, a good to excellent result can be achieved [4]. Since anatomically correct reconstruction is the key element for a satisfactory functional and clinical outcome [10, 11], the question arises whether the outcome can be improved by using other methods or techniques. Recent studies have shown that the use of three-dimensional (3D) planning and assisted osteotomies helps to perform even complex osteotomies accurately [12]. Therefore, measurement techniques based on CT-reconstruction 3D models of bone anatomy have been developed [13]. These techniques have been improved in recent years, and use a mirrored model of the anatomy of the contralateral bone as a template for the normal anatomy. Comparison of the 3D reconstructed models facilitates understanding of the deformity and is used to create patient-specific templates [12, 14, 15]. This method could improve the accuracy of ankle anatomy reconstruction. To the best of our knowledge, there is no method currently available for reconstructing the distal fibula based on contralateral 3D registration. Furthermore, it is unclear which anatomical landmarks or segments are the most accurate for this purpose. The aim of this study was to analyze the accuracy of a 3D registration algorithm using different segments of the contralateral side to restore the distal fibula. Anatomical differences between the two sides and patient-specific demographic characteristics were examined with respect to fibula restoration accuracy. We hypothesized that by using distinct anatomical landmarks [16, 17], the distal fibula could be restored more accurately.

## Methods

Ninety-six lower leg cadavers provided by the Institute of Forensic Medicine of the University of Zurich and previously analyzed in former studies [13, 16], were included in the study. The inclusion criterion was an existing CT assessed by the first author that included the entire tibia and fibula of both sides. Exclusion criteria comprised radiologically apparent previous trauma, surgery, advanced degenerative changes, or deformity of the tibia or fibula. Due to the well-known radiological criteria for osteoarthritis and clearly identifiable posttraumatic deformities, no inter-reader reliability was performed.

**Fig. 1:**
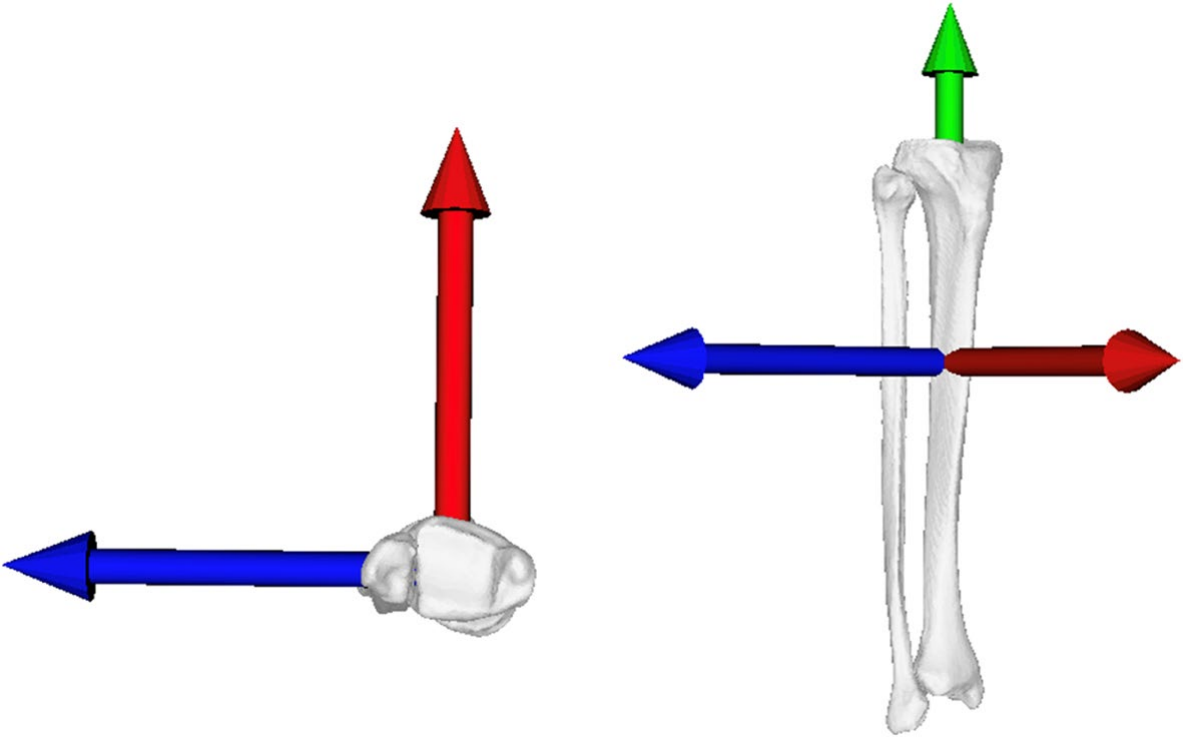
Definition of the anatomical coordinate system. The origin was located in the geometric center of the tibia, the X-axis (red) points from posterior to anterior, the Y-axis (green) points from distal to proximal, and the Z-axis (blue) points from medial to lateral

Thirty-four male and twelve female donors (two samples lacked gender information) were included with a mean age of 52 ± 17.7 years (range: 21 to 95 years). Mean weight was 83.1 ± 16.5 kg (range: 55 to 111 kg), and mean height was 176.2 ± 8.6 cm (range: 154 to 195 cm). High-resolution CT data were acquired using a Somatom Definition Flash CT scanner (Siemens®, Erlangen, Germany) with a slice thickness of 0.5 to 0.6 mm. 3D triangular surface models of 96 paired (48 left, 48 right) healthy tibiae and fibulae were created with manual threshold segmentation and region growing using MIMICS software (MIMICS Medical, Materialise NV, Leuven, Belgium). The bone models were imported into the surgical planning software CASPA (Balgrist CARD AG), developed in-house. To approximate the original distal fibula from the mirrored contralateral side, an iterative point proximity (ICP) algorithm [18, 19] was used to superimpose the mirrored contralateral model on the original model as described in previous studies [13, 17]. A 3D coordinate system was defined according to Wu et al. [20]; y-axis same direction vector as the anatomical tibial axis defined by an oriented bounding box (OBB) [21], z-axis: lateral, x-axis: anterior (Fig. 1).

### Definition of tibia and fibula segments for contralateral registration

**Fig. 2:**
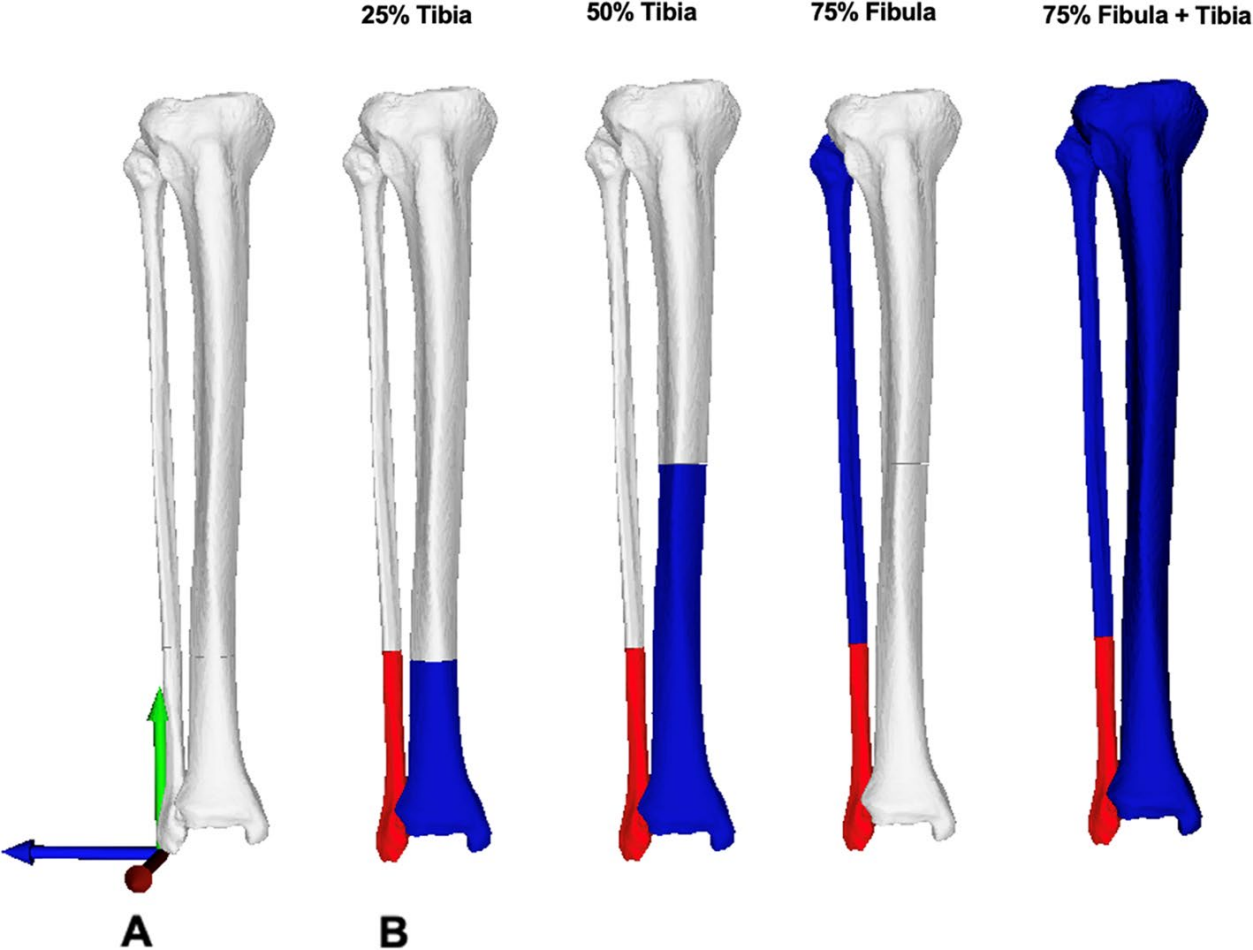
Definition of tibia and fibula segments for contralateral registration. The contralateral model was mirrored and four anatomical segments were defined and depicted from left to right: 25% tibia: including 25% of the tibia length; 50% tibia: including 50% of the tibia length; 75% fibula: including 75% of the fibula length; 75% fibula and tibia: including 75% of the fibula length and the complete tibia model

As segment selection and registration of anatomical structures potentially improve the accuracy of approximation to the premorbid anatomy [17], four different lower limb segments were defined to restore the distal fibula, excluding the possibly deformed distal 25% of the fibula. The contralateral lower leg model was mirrored, and four anatomic segments were defined (Fig. 2).

- *25% tibia*: the segment was defined as 25% of the distal tibial length.
- *50% tibia*: the segment was defined as 50% of the distal tibial length.
- *75% fibula*: the segment was defined as 75% of the proximal fibula length.
- *75% fibula and tibia*: the segment included the whole tibia model and 75% of the proximal fibula length (Fig. 2).

The surface registration algorithm for superimposing the mirrored contralateral models on the original model was performed for all four defined segments of the tibia and fibula with specified length, as described above.

### Accuracy of distal fibula restoration

**Fig. 3:**
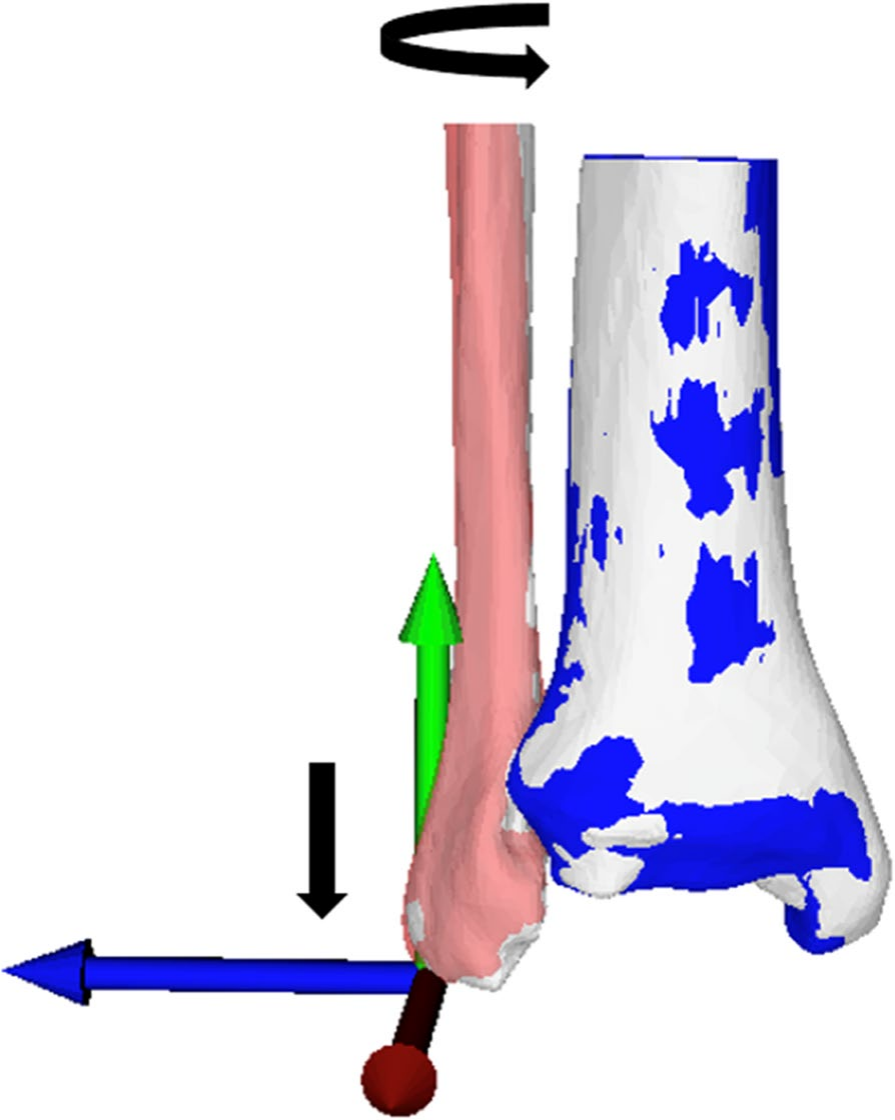
25% tibia segment (blue, contralateral mirrored) to restore the distal fibula. The blue tibia is superimposed to the white ipsilateral tibia using the ICP method. Red fibula = target ipsilateral fibula; white fibula = contralateral mirrored fibula. Using the 25% tibia segment as a reference result in a distalization of the white contralateral fibula by 0.5 mm and an internal rotation of − 0.6°

Translation and rotation of the distal contralateral fibula were measured in comparison to the original distal fibula and reported as errors. Translation was measured in mm (positive values indicate lengthening of the distal fibula, negative values indicate shortening), and rotation was measured in degrees (positive values indicate external rotation, negative values indicate internal rotation) around the anatomical axis (y-axis) (Fig. 3). Furthermore, the 3D distance (Euclidean distance) between the target ipsilateral distal fibula and the contralateral mirrored distal fibula was calculated. Similarly, the 3D angle (Euler’s angle) was calculated between the target ipsilateral distal fibula and the contralateral mirrored distal fibula to quantify positional deviation. In addition, the median absolute error of the distal fibula (translation, rotation, Euclidean distance, Euler’s angle) was defined for each segment. Bilateral models without pathology were used for the calculations. Accordingly, the error would be 0 mm or 0° if the anatomy were perfectly reconstructed.

### Measurement of the tibia and fibula length

The length of the tibia and fibula model was defined by the OBB [13]. Side-to-side differences are reported as median absolute differences.

Due to the highly standardized definition of the surfaces and the largely automated measurement procedure, no inter- and intra-reader reliability was performed.

#### Statistical analysis

Statistical analysis was conducted with SPSS software v26.0 (IBM, New York, USA).

The Shapiro-Wilk test was applied to test the data for normal distribution. The variables are reported as median and range. As the data were not normally distributed, the Friedmann’s test (nonparametric ANOVA for related samples) was applied to study between-level differences. Outcomes with significant differences were further analyzed in a pairwise comparison using Wilcoxon signed rank tests. The respective *p*-values were Bonferroni-corrected as applicable. To identify patient-specific factors, including age, sex, BMI and tibia and fibula side-to-side differences, associated with the outcomes of interest, a stepwise linear regression model was applied. In this analysis, missing values (weight and height information were missing in nine donors; gender information was lacking in two cases) were not taken into account, and the corresponding cases were excluded from the analysis. The alpha level was set at 0.05, and all *p*-values were two-tailed.

## Results

Considering all four segments, an overall median translation error of 0.1 mm (− 5.4–5.6), a median rotation error of 2.1° (− 16.5–26.9), a median Euclidean distance of 2.9 mm (0.1–11.1) and a median Euler’s angle of 4° (0–26.9) were calculated for the accuracy of distal fibula restoration.

### Accuracy of distal fibula restoration using the four anatomic segments

**Fig. 4:**
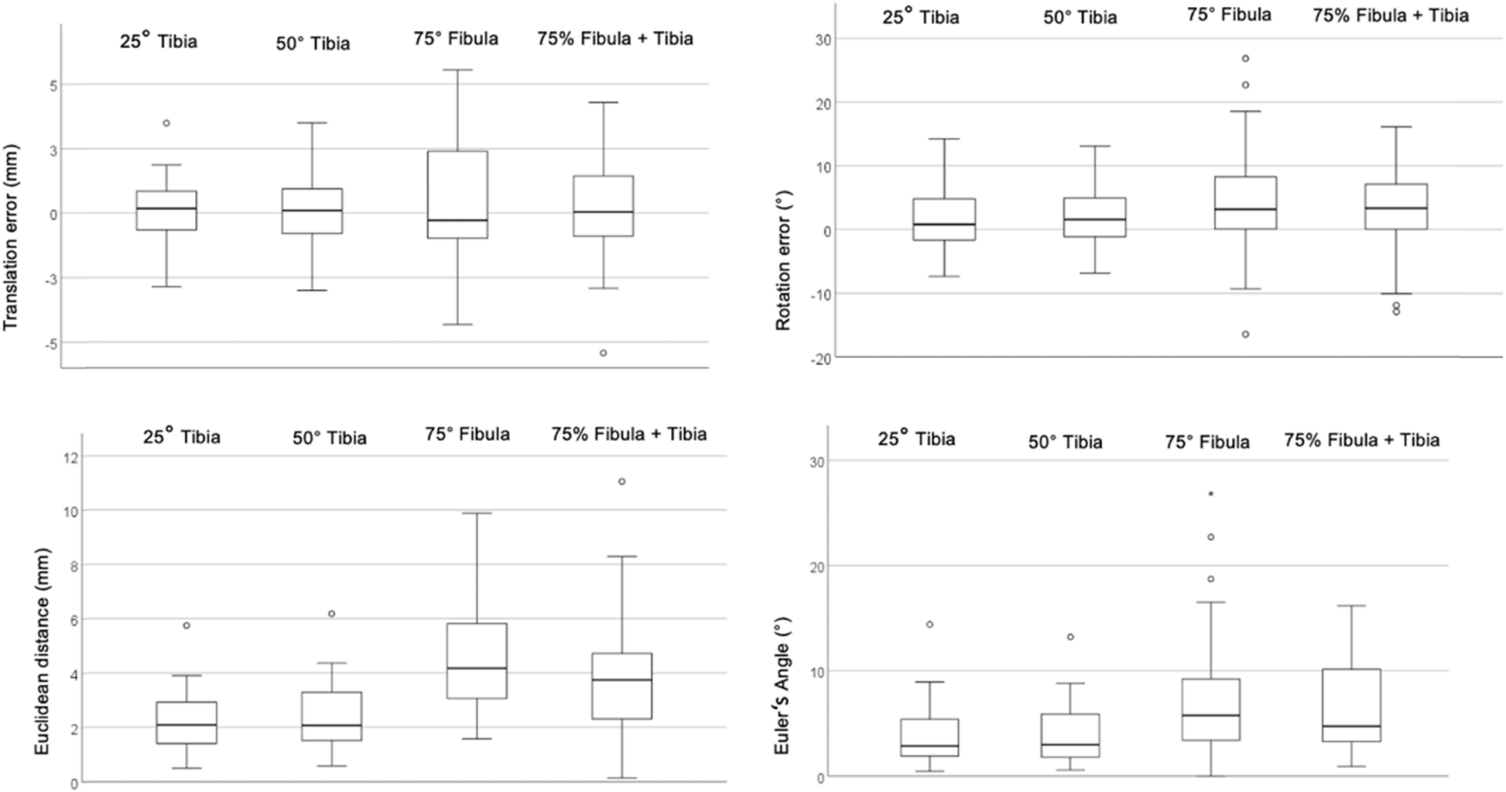
Median errors for all segments. Y-axis depicts median translation error, median rotation error, median Euler’s angle and median Euclidean distance for all four segments; IQR (box), range (whiskers) and outliers (points)

The translation error did not differ significantly between segments (Table 1). The rotational error (internal/external rotation), Euclidean distance, and Euler’s angle were highest if the proximal 75% of the fibula were used, decreased if 75% of the proximal fibula and the whole tibia or 50% of the tibia alone were used and were significantly smaller if 25% of the distal tibia were used (Table 1, Fig. 4). Rotational error was significantly smaller when the 25% distal tibia and 50% distal tibia were used compared with the union, *p* < 0.016 and *p* < 0.014, respectively. Similarly, Euclidean distance and Euler’s angle were significantly reduced when the 25% or 50% distal tibial segment, rather than the proximal 75% of the fibula or the union, was used for restoration (*p* < 0.001).

**Table 1:** Accuracy of the distal fibular restoration based on the four segments

	25% Tibia	50% Tibia	75% Fibula	75% Fibula + Tibia	*p*-value^‡^
Translation error (mm)	0.2 (−2.8–3.5)	0.1 (−3–3.5)	−0.3 (− 4.3–5.5)	0 (−5.4–4.3)	.498
Rotation error (°)	0.8 (−7.4–14.2)	1.6 (− 6.8–13.1)	3.2 (− 16.5–26.9)	3.3 (− 12.9–16.1)	**.003**
Euclidean Distance (mm)	2.1 (0.5–5.7)	2.1 (0.6–6.2)	4.2 (1.6–9.9)	3.7 (0.1–11.1)	**<.001**
Euler’s Angle (°)	2.9 (0.5–14.4)	3 (0.6–13.2)	5.8 (0–26.9)	4.7 (0.9–16.2)	**<.001**

^§^Values in median and ranges ()

^‡^Friedmann’s test

In the linear regression model, no patient-specific factors could be identified that influenced rotational or translational errors.

## Discussion

The most important finding of our study is that the contralateral tibia and fibula can be reliably used to restore the distal fibular anatomy. The inclusion of anatomical landmarks of the fibular notch and malleolar colliculi in the registration protocol reduced both rotational and translational error and led to a more accurate approximation of the distal fibula. Therefore, the hypothesis can be confirmed.

By referring to anatomical landmarks, the premorbid anatomy can be restored very accurately, as has already been shown in previous studies [16, 17]. To date, radiological parameters such as talar inclination, talocrural angle, or bimalleolar angle [22–24] are used for both preoperative planning of corrective osteotomies and postoperative evaluation. Even small side-to-side differences of a few degrees have been associated with a poorer clinical and functional outcome [10, 11], emphasizing the importance of exact restoration of the premorbid anatomy. Bilateral 3D registration of the distal tibia appears to be the method best suited for 3D approximation of the distal fibular anatomy. Prominent landmarks of the distal tibia, such as the anterior medial colliculus, the posterior medial colliculus, the tuberculum Tillaux-Chaput, and the tibial plafond, allow the models of both sides to be superimposed in such a way that the error of translation (shortening/lengthening) and rotation (internal/external rotation) is smaller than with a larger number of irrelevant reference points or overall anatomy. The reasons are probably the length independence of the distal tibia and the absence of antero-posterior as well as lateral displacements, which are more likely to be encountered when large bony segments are used. This explains why the smaller segment of 25% of the distal tibia had significantly less error compared to other, longer segments. In our opinion, this is a simple approach to follow, requiring only a preoperative CT scan of the contralateral ankle including the tibial segment approximately ten centimeters proximal to the joint line. Of course, correct preoperative planning alone does not improve the clinical outcome. However, if the planned correction can be implemented with surgical precision, e.g. by using patient-specific templates, the outcome may be positively influenced. Surgical precision hardly allows to exceed the stated accuracy of the error presented here of 0.2 mm and 0.8° for translation and rotation, respectively. Therefore, the stated accuracy should be acceptable in terms of clinical relevance.

Adaptation of CT protocols and automation of segmentation protocols has led to a reduction in radiation exposure and cost [25, 26]. In the future, further improvements are likely; thus, the contralateral registration method appears reliable to further improve outcomes of fibular corrective osteotomies.

The main limitation of the present study is that the 3D registration method depends on a healthy contralateral anatomy, therefore a preoperative assessment can only be applied in healthy bone. For this reason, several segments were chosen and analyzed to allow registration despite the presence of deformity, osteoarthritis, previous contralateral arthrodesis, or total ankle arthroplasty of the contralateral side. However, the restoration errors could be higher than in the results reported here if only a smaller segment (< 25%) is available for fibula registration, e.g. in the case of a far distal fracture. A combination with area and volume measurements could possibly reduce a larger error [27] and could be investigated in a further study. Furthermore, information about the medical history of the cadavers was limited. Therefore, specimens with signs of deformities, previous surgeries or fractures were excluded.

## Conclusion

3D registration of the contralateral tibia and fibula reliably approximated the premorbid anatomy of the distal fibula. Registration of the 25% distal tibia, including distinct anatomical landmarks of the fibular notch and malleolar colliculi, restored the anatomy with increasing accuracy, minimizing both rotational and translational errors. This new method of evaluating malreductions could reduce morbidity in patients with ankle fractures.

## Data Availability

The datasets used and/or analysed during the current study are available from the corresponding author on reasonable request.

## Abbreviations

3D: Three-dimensional
CT: Computer tomography
OBB: Oriented bounding box
ICP: Iterative closest point
